# Fatal poisoning by ingestion of a self-prepared oleander leaf infusion

**DOI:** 10.1007/s12024-020-00338-w

**Published:** 2020-11-25

**Authors:** Anna Carfora, Raffaella Petrella, Renata Borriello, Lucia Aventaggiato, Roberto Gagliano-Candela, Carlo Pietro Campobasso

**Affiliations:** 1 Department of Experimental Medicine, Forensic Toxicology Unit, University of Campania “L. Vanvitelli”, Via L. Armanni, 5, 80138 Napoli, Italy; 2 Department Interdisciplinary of Medicine (DIM), Forensic Toxicology Laboratory, University of Bari, Piazza Giulio Cesare, 11, 70124 Bari, Italy

**Keywords:** Poisoning, *Nerium oleander* leaves, Oleandrin analysis, Oleandrin/oleandrigenin in vitreous humor, Liquid chromatography-tandem mass spectrometry

## Abstract

An unusual case of poisoning by the ingestion of oleander leaves is reported. A 71 year old male laboratory technician committed suicide at home in this unusual manner. At the death scene a steel pan and other paraphernalia, used for the extraction of oleandrin and other cardiac glycosides from the leaves of the *Nerium oleander* plant were found.

Toxicological investigations for oleandrin, oleandrigenin, neritaloside, and odoroside were performed by LC–MS/MS on all biological samples (peripheral blood, vitreous humor, urine, liver, gastric contents) and on the yellow infusion found at the death scene.

In all samples, toxic levels of oleandrin were detected (blood 37.5 ng/mL, vitreous humor 12.6 ng/mL, urine 83.8 ng/mL, liver 205 ng/mg, gastric content 31.2 µg/mL, infusion 38.5 µg/mL). Qualitative results for oleandrigenin, neritaloside, and odoroside were obtained. Oleandrigenin was present in all tissue samples whereas neritaloside and odoroside were absent in the blood and vitreous humor but present in urine, liver, gastric content, and in the leaf brew.

The purpose of this study was the identification of oleandrin and its congener oleandrigenin, detected in the vitreous humor. The blood/vitreous humor ratio was also calculated in order to assess of the likely time interval from ingestion to death. According to the toxicological results death was attributed to fatal arrhythmia due to oleander intoxication. The manner of death was classified as suicide through the ingestion of the infusion.

## Introduction

*Nerium oleander* (or oleander) is an ornamental evergreen shrub belonging to the family Apocynaceae, widespread in the Mediterranean area, but also in subtropical and tropical regions. Oleander contains, in each of its parts (seeds, roots, leaves, flowers, fruits, branches, and stem), several cardiac glycosides (CGs), also defined as cardenolides [1].

Oleandrin is the most relevant toxin of the Oleander plant and it is the only one available as a pure standard [2]. The chemical formula of oleandrin is C_32_H_48_O_9_ and its potential cardiotoxicity has been well known since ancient times. CGs are secondary compounds found in plants and amphibians, widely distributed in nature with a potential cardiovascular action [3]. The most common CGs are digoxin, ouabain, oleandrin, and bufalin. Due to their similar chemical structure, CGs share well-known similarities in the mechanism of action, and their cardiovascular toxicity usually restricts their clinical use. Clinical signs are mostly represented by gastrointestinal, neurological, and cardiovascular problems. Episodes of emesis such as vomiting, nausea, and abdominal pain are common at the beginning of acute oleander intoxication as a physiological reaction to reduce toxin absorption. Other symptoms of CGs poisoning include diarrhea, tremors, visual disturbance, hyperkalemia, sinus bradycardia, and ventricular arrhythmia [3].

Oleander poisoning is mostly accidental in children and pet animals. It can also occur in livestock due to the ingestion of contaminated forage [4–6].

Accidental human exposure to the oleander and the intentional ingestion of natural preparations for medicinal purposes have been widely reported in South Asian countries (Sri Lanka, India). Very few cases have been reported in Europe, Australia, and the United States [7, 8]. Herbal products derived from the oleander are commonly suggested as "*harmless*" home-made treatments or medication because they are natural products. They are used for a broad range of treatments such as slimming, muscle enhancement, erectile dysfunction, malaria, epilepsy, psoriasis, herpes, eczema, and thyroiditis, and for cancer treatment [9, 10]. Hence, lipid soluble CGs such as oleandrin have been found to be useful as anticancer agents against some cancer cell lines [11]. However, the simultaneous use of the oleander both as a therapeutic drug and as a natural remedy could lead to an inappropriate use of this plant and it is more likely to produce an increase of poisonings or fatal cases [12, 13]. A fatal event after drinking herbal tea prepared from oleander leaves has been reported [14]. Boiling or drying the plant leaves does not inactivate the CGs.

The intake of oleander leaves, or the infusions obtained from various parts of this plant is uncommon for suicidal purposes and only a few cases are reported in the literature [13, 15, 16]. Different analytical methods for the identification and quantification of oleandrin are available. These include techniques from the rapid digoxin and digitoxin immunoassay for clinical purposes [17], to liquid chromatography-electrospray tandem mass spectrometry (LC–MS/MS), which is the best analytical approach to oleander poisonings of forensic interest [1, 2, 18]. Although in fatal cases of forensic interest, only oleandrin is almost always found in biological samples, and no information about the presence of other CGs (such as oleandrigenin, neritaloside, and odoroside, present in hot water-based extract f the oleander plant) are available.

The aim of the study was the identification of oleandrin and its congener oleandrigenin in tissue samples taken from a fatal poisoning through the ingestion of an infusion prepared from oleander leaves and other parts of the plant (*Nerium oleander*). LC–MS/MS analysis for oleandrin, oleandrigenin, neritaloside, and odoroside was performed both in biological matrices and on the infusion sampled at the death scene. The blood/vitreous humor ratio for oleandrin was also calculated to assess of the likely time interval from ingestion to death.

## Case Report

**Fig. 1:**
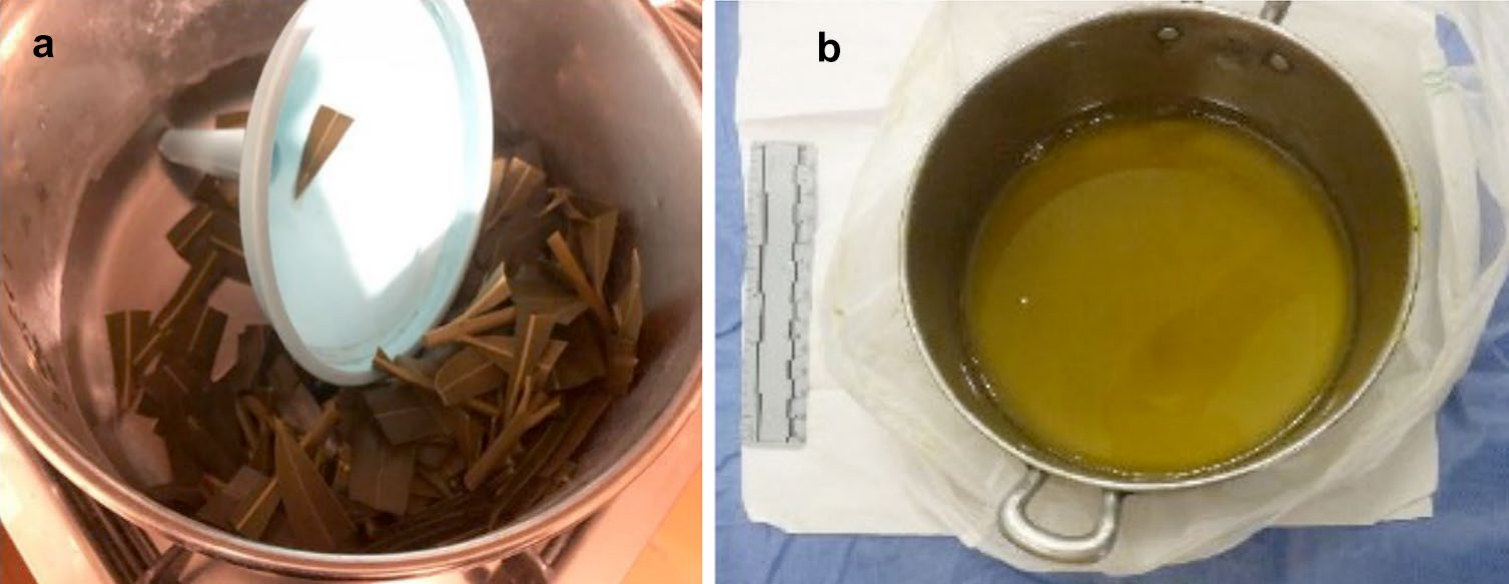
Items found at the death scene: (**a**) the steel pan containing 256.8 g of dark green leaves, small pieces of stem, and a plastic funnel; (**b**) the second smaller steel pan containing 400 mL of yellow fluid infusion

A 71 year old white male, who had worked as a laboratory technician in the past, was found dead at home. During the scene survey, a steel pan medium size, closed with its cover and sealed with packaging tape, was found near the body. A small piece of white sellotape was on the cover, with the following hand-written note on it: “*Poison. Wash pan and funnel carefully or throw everything away*”. Elongated dark green leaves weighing 256.8 g, along with small pieces of stem and a plastic funnel were found within the pan (Fig. 1A). A second steel pan, smaller than the first one (Fig. 1B), and a plastic bottle were also present at the scene, containing respectively 400 mL and 100 mL of a yellow fluid infusion. On the bottle, a small piece of white sellotape was present with a second hand-written note: “*Poison*”. An empty glass was found close to the bottle, both of which were on the bedside table close to the dead body.

No relevant autopsy findings were observed except for multi-organ congestion, which is common in fatal poisoning. Visceral congestion was observed in the liver, brain, and lungs. The lungs also showed some histologically evident hemorrhagic pulmonary edema. Signs of interstitial and intra-alveolar pulmonary edema were observed and represented by the dilatation of alveolar spaces filled by acellular edema fluid along with alveolar hemorrhages and thickness of the alveolar walls due to capillary hyperemia or marked congestion of vessels. No other findings of injury or disease were observed. A few milliliters of brown fluid were present in the stomach. Samples of peripheral blood, vitreous humor, urine, liver, and gastric content were collected for toxicological analysis.

## Toxicological analysis and results

The study was performed in accordance with the ethical standards as laid down in the 1964 Declaration of Helsinki and its later amendments.

LC–MS/MS analysis was applied to detect the active substance oleandrin and the related CGs (oleandrigenin, neritaloside, and odoroside) in all the available biological samples (peripheral blood, vitreous humor, urine, liver and gastric content) and in the fluid infusion of oleander leaves.

Aliquots of 1mL of the gastric content and of the oleander leaf infusion were filtered and progressively diluted with 0.5% formic acid in water solution (v/v) (eluent A).

Aliquots of 1mL of peripheral blood, urine, vitreous humor, and 1g of homogenized liver were extracted with 3 mL of Ethyl Acetate (1:3) mixing the samples vigorously for 10 min, as described by Zhai et al. [19]. The mixture was centrifuged at 6000 rpm for 5 min and a filtered aliquot of the clear extract was transferred into vials for the LC–MS/MS autosampler.

Moreover, in order to accomplish a systematic toxicological analysis, urine and blood were also analyzed by gas chromatography coupled with mass spectrometry (GC–MS) for different classes of drugs of abuse and pharmaceuticals, following the procedures reported by Carfora et al. [20]. Blood was tested for alcohol, and for other volatile substances, by headspace gas chromatography (GC/HS).

## LC–MS/MS analysis

The LC–MS/MS analyses were performed by a triple quadrupole mass spectrometer (AB Sciex 3200, Carlsbad, USA) connected with a Liquid Chromatography from Agilent 1200 equipped with a Zorbax DB-C18 column (4.6 × 50 mm, particle size 1.8 µm).

Chromatographic separation was achieved by using a gradient of two different mobile phases (eluent A: 0.5% formic acid in water (v/v), and eluent B: 0.5% formic acid in acetonitrile (v/v)). The gradient program started with 5% B at 0–0.2 min, increasing to 100% at 5 min, held until 7 min. Subsequently, the B content was reverted to 5%. The injection volume was 10 mL. The total run time was 10 min and the flow rate was 0.9 mL/min.

The retention time for oleandrin was 4.1 min and for neritaloside, odoroside and oleandrigenin were 3.45, 3.62, and 3.90 min, respectively. MS data were acquired in electrospray ionization (ESI) positive mode, using the following Turbo Ion Spray source conditions: temperature, 400 °C; curtain gas, 30 (arbitrary units); GS1 and GS2, 50; CAD gas pressure, low; ion spray voltage, 5500.

Acquisition parameters were optimized while 1 mg/mL oleandrin standard at 10 mL/min was infused into the mobile phase. Monitored ions for oleandrin were the precursor ion of *m/z* 577 to 373.0, 433.0, and 355 product ions. For the other glycosides, the monitored ions were *m/z* 593 to 373 product ions for neritaloside, *m/z* 433 to 373 for oleandrigenin and *m/z* 535 to 375 for odoroside [21].

## Standard solutions, calibration curves and toxicological results

Oleandrin standard was purchased from Sigma Chemical Co. (St. Louis, MO) (> 95% purity). A stock solution of 1000 µg/mL was made in methanol. A five-point calibration curve of oleandrin in the range 10-250 ng/mL (in eluent A) was prepared and analyzed by LC–MS/MS methodology as described above.

In order to quantitate oleandrin in biological matrices and in the oleander infusion, a five-point calibration curve was prepared trough the standard addition of oleandrin at 10, 50, 100, 200, and 250 ng/mL to each sample, adopting the most appropriate dilution for gastric content and the leaf infusion, before the pretreatment or extraction. Oleandrin limit of detection (LOD) was 1 ng/mL and the lower limit of quantification (LLOQ) was 2 ng/mL.

In order to test the recovery and accuracy of the applied methods, three known concentrations of the standard oleandrin (10–50-200 ng/mL) were spiked to drug-free whole blood and urine and assayed in three replicates. After the extraction and LC–MS/MS analysis, a mean recovery and accuracy of 88% and 92% respectively were calculated.

As neritaloside, oleandrigenin and odoroside were not commercially available, for these glycosides only qualitative analyses were performed.

**Fig. 2:**
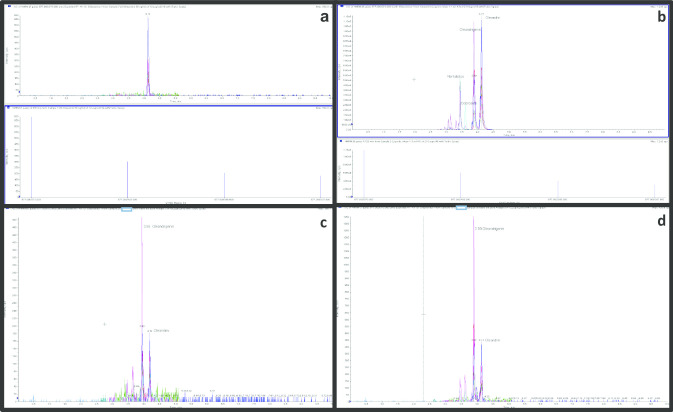
LC–MS/MS chromatograms for oleandrin in standard solution (**a**), for oleandrin, neritaloside, odoroside and oleandrigenin in the liquid of infusion (**b**), for oleandrin and oleandrigen in vitreous humor (**c**), and peripheral blood (**d**) extracts. For each graph, Y-axis: Intensity abundance (cps) and X-axis: acquisition time (min)

Toxicological quantitative results for oleandrin and relative qualitative data (cps on peak height) for the presence of neritaloside, odoroside, and oleandrigenin, are shown in Table 1 and in Fig. 2. The systematic toxicological analysis (STA), applied to all biological samples, did not detect the presence of other xenobiotic substances.

**Table 1:** Oleandrin concentration levels and qualitative results of the other cardiac glycosides (CGs) of the *Nerium Oleander*, in biological samples and in the oleander leaves infusion

Samples	Oleandrin*	Oleandrigenin** Intensity cps	Neritaloside** Intensity cps	Odoroside** Intensity cps
Peripheral blood	37.5 ng/ml	1356	< 100	< 100
Vitreous humor	12.6 ng/ml	487	< 100	< 100
Urine	83.8 ng/ml	3974	368	< 100
Liver	205 ng/mg	5248	200	160
Gastric content	31.2 µg/ml	1.4 × 10^5^	3.8 × 10^4^	1.6 × 10^4^
Fluid infusion	38.5 µg/ml	1.3 × 10^5^	4.5 × 10^4^	2.0 × 10^4^

*LOD 1 ng/mL, *LLOQ *2 ng/mL

**Considering the noise/signal ratio as a threshold level 100 cps

## Discussion

The detection of oleandrin in cadaveric biological samples revealed fatal concentrations in peripheral blood (37.5 ng/mL). According to the literature, oleandrin blood levels of about 1–2 ng/ml are regarded as toxic [1, 13], and blood concentrations of 9.8–10 ng/mL have been detected in fatal acute poisoning cases [12, 18].

In the present case study, oleandrin and oleandrigenin were also detected in vitreous humor (Fig. 2c) and the blood/vitreous humor concentrations ratio for oleandrin was 2.97. According to the distribution time from blood to vitreous humor [22], the high blood/vitreous ratio suggests a rapid death, which occurred shortly after the intake because the equilibrium between the two matrices was not yet reached. This is also consistent with the observed hemorrhagic pulmonary edema, a common histological finding in acute heart failure due to ventricular arrhythmia by cardiotoxic substances like oleandrin [23]. In fact, oleandrin specifically inhibits the Na^+^/K^+^ ATPase of cardiomyocytes, resulting in a positive inotropic effect (fast and strong heart contraction) due to hydro-electrolytic imbalance represented by the accumulation of extracellular K^+^ and intracellular Na^+^ and Ca^2+^ [3, 8, 23]. Unfortunately, no other data for oleandrin and oleandrigenin in vitreous humor were found in the literature, including a recent review reporting the distribution of 106 xenobiotics in vitreous humor samples from more than 300 case reports [24]. It is also interesting that oleandrigenin, the aglycone metabolite of oleandrin, was detected in all available biological samples, in quantities ranging between the levels found in the gastric contents and those in the yellow fluid infusion. A decreasing order of these levels goes from the liver through urine and peripheral blood up to vitreous humor. Neritaloside and odoroside, although present in the yellow fluid infusion and in the gastric contents, were not found in peripheral blood and vitreous humor, but was found in smaller concentrations in the other samples, compared to the oleandrigenin and oleandrin. We hope our findings are useful for the evaluation of future cases.

Episodes of adult poisoning through plants can be either intentional or accidental. Accidental oleander poisoning occurs rarely, but educational programs would be appropriate to avoid misunderstandings about the dangerousness of the *Nerium oleander* plant. Intentional ingestion of parts of the oleander plant frequently leads to severe symptoms and, sometimes, death. In fact, only people such as the adult male of the present case study, who are aware of the cardiotoxicity of the oleander glycosides, are able to use these substances for their purposes. He was a laboratory technician who knew the properties of the Oleander plant very well as was clearly indicated by the hand-written notes found on the items used to prepare the oleander infusion. Such notes demonstrate his awareness of the potential toxicity of the oleander extract, and his suicidal intensions, which are often attributable to particular categories of retired workers [25, 26].

Home-made natural infusions following recommendations found on web sites can be dangerous. Promoting a wider awareness of the potential toxicity induced by the intake of parts of *Nerium oleander* plant may be of help to reduce the incidence of poisonings.

Toxicological results, performed on samples collected during the autopsy and at the death scene, supported the diagnosis of self-poisoning through the ingestion of the oleander infusion. In this fatal case, the main cardiac glycoside of *Nerium oleander* (oleandrin) was detected at toxic levels in all available biological samples. The presence of oleandrin and its congener oleandrigenin was also detected in the vitreous humor. In similar events, the blood/vitreous humor ratio can be useful for the estimation of the survival time interval, which was short in the present case study.

## Key points

1. Ingestion of oleander leaf infusion can be accidental but also intentional.

2. The potential cardiotoxicity of oleandrin is well known.

3. Oleandrin and oleandrigenin can be detected in the vitreous humor by LC–MS/MS.

4. A high blood/vitreous humor ratio can suggest a short time interval from ingestion to death.
